# A Weighted Polygenic Risk Score Using 14 Known Susceptibility Variants to Estimate Risk and Age Onset of Psoriasis in Han Chinese

**DOI:** 10.1371/journal.pone.0125369

**Published:** 2015-05-01

**Authors:** Xianyong Yin, Hui Cheng, Yan Lin, Nathan E. Wineinger, Fusheng Zhou, Yujun Sheng, Chao Yang, Pan Li, Feng Li, Changbing Shen, Sen Yang, Nicholas J. Schork, Xuejun Zhang

**Affiliations:** 1 Institute of Dermatology, Department of Dermatology, The First Affiliated Hospital, Anhui Medical University, Hefei, Anhui Province, China; 2 Key lab of Dermatology, Ministry of Education, State Key Lab of Dermatology Incubation Center, Anhui Medical University, Hefei, Anhui Province, China; 3 Key Lab of Gene Resource Utilization for Complex Diseases, Hefei, Anhui Province, China; 4 Collaborative Innovation Center for Complex and Severe Dermatosis, Anhui Medical University, Hefei, Anhui Province, China; 5 Scripps Health, La Jolla, California, United States of America; 6 The Scripps Translational Science Institute, La Jolla, California, United States of America; 7 Department of Molecular and Experimental Medicine, The Scripps Research Institute, La Jolla, California, United States of America; 8 Human Biology, J. Craig Venter Institute, La Jolla, California, United States of America; University of Texas, UNITED STATES

## Abstract

With numbers of common variants identified mainly through genome-wide association studies (GWASs), there is great interest in incorporating the findings into screening individuals at high risk of psoriasis. The purpose of this study is to establish genetic prediction models and evaluate its discriminatory ability in psoriasis in Han Chinese population. We built the genetic prediction models through weighted polygenic risk score (PRS) using 14 susceptibility variants in 8,819 samples. We found the risk of psoriasis among individuals in the top quartile of PRS was significantly larger than those in the lowest quartile of PRS (OR = 28.20, P < 2.0×10^-16^). We also observed statistically significant associations between the PRS, family history and early age onset of psoriasis. We also built a predictive model with all 14 known susceptibility variants and alcohol consumption, which achieved an area under the curve statistic of ~ 0.88. Our study suggests that 14 psoriasis known susceptibility loci have the discriminating potential, as is also associated with family history and age of onset. This is the genetic predictive model in psoriasis with the largest accuracy to date.

## Introduction

Psoriasis is a common chronic inflammatory immune-mediated disease that affects ~ 3% of the world’s population [1]. Psoriasis is characterized by red skin scales and plaques, and vascular remodeling [1]. It is widely accepted that genetic predisposition and environmental factors (such as excessive alcohol consumption, tobacco smoking, etc.) contribute to the risk and development of psoriasis [1, 2]. In recent years, a number of common variants have been found to be associated with psoriasis, mostly through GWASs in diverse populations [3–12]. Although these studies have greatly expanded the understanding of genetic and biological mechanisms for psoriasis, most of the associated genetic loci to date confer a small or moderate effect on psoriasis risk and collectively only explain a small fraction of the heritable component of psoriasis [13]. Therefore, it remains unclear whether these genetic discoveries can be used to create a sufficiently accurate predictive model for psoriasis risk or be used to subdivide psoriatics into more homogenous categories for treatment. Accurate risk prediction could help improve clinic consulting and enable targeted or ‘personalized’ preventive interventions, such as altering lifestyle or prescribing unique pharmacological agents.

Several predictive models incorporating GWAS-implicated variants have been built for human complex diseases [14–19]. Among them, a model combining 10 psoriasis risk loci improved the ability to discriminate psoriatics from non-psoriatics with an area under the curve (AUC) statistic achieving a value of 0.72, and it was found that their weighted risk score was a statistically significantly better discriminator than a model that included only individual single nucleotide polymorphism (SNP). They also found that their risk score was associated with early onset of disease and a positive family history [19].

We have conducted the first GWAS and several follow-up studies for psoriasis in Han Chinese population, identifying ~14 common variants with genome-wide significance [10, 12, 20, 21]. In this study, we sought to create predictive models using these 14 variants and to evaluate their discriminatory performance in two independent psoriasis cohorts of Han Chinese.

## Materials and Methods

### Study design and Participants

We applied a two-stage design (S1 Fig). We enrolled 4,541 psoriasis cases and 4,278 healthy controls through a consortium effort in China, consisting of 3,805 cases and 3,542 controls in the initial stage, and 736 cases and 736 controls in the independent validation stage. The samples in the initial stage have experienced our previous original GWAS and following up studies, while in the independent validation stage, none samples did. All the individuals used in the analysis were not closely related to each other in the initial cohort and validation cohort. All participants were diagnosed by at least two dermatologists. After a full clinical checkup, clinical and additional demographic information was collected using a previously developed structured questionnaire [12]. All controls were matched based on age, gender and geographic region, and they were clinically assessed as being without psoriasis, other autoimmune disorders, systemic disorders or a family history of autoimmune disorders (including first-, second- and third-degree relatives). To explore the impact of environmental factors in disease’s prediction, we collected alcohol abuse status and classified individuals into ever-drinking and never-drinking respectively (the latter including individuals whose alcohol intake less than 170 g/week) [22]. It was noted that the environmental information was not available for the samples in our validation stage. Written informed consent was given by all participants. The study was approved by the Institutional Ethics Committee of Anhui Medical University and was conducted in accordance with the Declaration of Helsinki principles.

### SNP selections and Genotyping

We chose the 14 SNPs previously identified for psoriasis with genome-wide significance in Han Chinese population (S1 Table). All individuals in the initial stage were genotyped with the Illumina Human 610-Quad BeadChips or Sequenom Mass Array system as described previously [10, 12, 20, 21]. We filtered 184 cases and 192 controls with missing genotypes.

In the validation study, twelve SNPs were subjected to Sequenom Mass Array system and other two (rs1265181 and rs7007032) were genotyped using ABI Taqman SNP genotyping arrays according to the manufacturer’s recommendations. The quality control procedures were implemented with following criteria: call rate > 90% per sample, genotyping rate > 90% per SNP and Hardy-Weinberg Equilibrium P > 10^–3^ in controls. Assuming each independent SNP confers an additive effect to the risk of psoriasis, all 14 SNPs passed quality controls and then were subjected to the single variant association test using standard chi-square analysis in Plink 1.07.

### Polygenic risk score calculation

A PRS was calculated as a weighted sum of the number of risk alleles possessed by an individual, in which the weight was taken as the natural log of the odds ratio (OR) associated with each individual SNP. We used published ORs from our previous studies to calculate PRS for both initial and replication stages (S1 Table) [10, 12, 20, 21]. The psoriasis associated effect size of alcohol drinking was taken from a recent meta analysis (S2 Table) [23]. We generated three different PRSs that included: 1.13 non-HLA SNPs (SNP PRS); 2.1 HLA SNP (HLA PRS) and 3.all 14 SNPs together (SNP-HLA PRS). In order to compare the discriminatory ability of the three genetic PRSs, we also built a 4th predictive model that included all SNPs and alcohol consumption (SNP-HLA-Drink). All individuals were categorized into four PRS groups (Group 0, 1, 2, 3) based on the quartiles of PRS values among healthy controls. Logistic regression was used to calculate the OR and associated p value assessing the relationship between psoriasis status and each PRS group with age and gender taken as covariates.

### Compare model classification abilities

We constructed receiver operating characteristic (ROC) curves and measured the AUC to evaluate the discriminatory ability of each model in the PRS analysis. AUCs were evaluated and compared using DeLong’s methods in R package pROC.

### Cox proportional hazards test

To investigate the impact of PRS, PRS group, family history and alcohol drinking on the age onset in our psoriasis cases in the initial stage, we applied a Cox proportional hazards test using age and gender as covariates. To further demonstrate associations between significant factors and psoriasis age of onset, we explored Kaplan-Meier estimates of the cumulative risk for cases. Testing of differences in Kaplan-Meier curves across groups was pursued with Log-rank statistics. All statistics were implemented in R 3.0.1.

## Results

### Summary statistics

We applied a two-stage design strategy (S1 Fig). In the initial stage, 3,621 patients and 3,350 controls were included. All these samples have been already used in our previous psoriasis original GWAS. Characteristics of our study sample are summarized in Table 1. The cases and controls were matched by gender and age. There were 31.34% cases observed with positive family history of psoriasis. In addition, 3,190 participants were available with alcohol abuse status (S3 Table). We tested the association between alcohol abuse and psoriasis in our sample and found that it was highly statistically significant (OR = 2.49, P < 2.16×10^–16^).We also tested the association between each of the 14 SNPs and psoriasis (S1 Table). The effective allele frequencies and ORs were generally similar to our previously published data (S1 Table). Any discrepancies between our current allele frequency estimates, ORs, etc. and our previously published estimates probably represent normal sampling variation and differences in sample size.

**Table 1:** The characteristics summary of samples in the initial stage.

	Case	Control
**No.**	3621	3350
**Male (%)**	2027(55.98%)	2062(61.55%)
**Age**		
mean(min-max)	30.46(3–80)	29.88(3–81)
s.d.	11.99	10.96
**Age onset**		
mean(min-max)	21.31(1–39)	—
s.d.	8.69	—
**Family History (%)**	1135 (31.34%)	—

s.d.: standard deviation. mean: the mean value of age/age onset. min/max: the minimal/maximal vale of age/age onset.

To validate the genetic predictive model, we carried out a replication study by using the 14 SNPs in an independent cohort. After quality control, there were 712 cases and 723 controls remained. These samples have not experienced the previous GWAS. Among the 14 SNPs, seven were validated with at least nominal significance (P<0.05) (S4 Table). It was shown that we had insufficient statistic power (Power <40%) for the other seven SNPs at nominal significance P < 0.05 (S4 Table).

### PRS and the risk of psoriasis

In the initial stage, the SNP-HLA PRS in cases was comparable with that in controls (mean: 2.436, 95% quartiles: 1.634–2.559 in controls; mean: 4.648, 95% quartiles: 4.335–5.240 in cases, S2 Fig). In our sample, 86.94% cases were grouped into the top quartile of PRS (PRS > 2.56), while only 3.15% were categorized as bottom quartile of PRS (PRS ≤ 1.64) (S4 Fig). It was indicated the continuous PRS increased the risk of psoriasis (β = 1.03, P<2×10^–6^). We explored the distribution of risk effect size across four distinct SNP-HLA PRS groups. We found that the risk of psoriasis increased significantly along with PRS (Table 2 and S6 Fig). Compared with individuals in the bottom quartile of PRS, individuals with the top quartile of PRS had a ~ 28-fold risk of psoriasis (OR = 28.20, P < 2.00×10^–16^), suggesting that these 14 SNPs collectively confer a dominant risk for psoriasis. In PRS logistic regression model, it was shown that these 14 SNPs totally covered 11.6% phenotypic variation. In the independent validation cohort, the PRS distribution pattern and significant PRS-psoriasis association were achieved with consistent evidences (S3, S5 and S7 Figs)

**Table 2:** The risk of psoriasis between 4 SNP-HLA PRS groups in the initial stage.

Polygenic risk score	No. Case	No. Control	OR(95%CI)	P
< = 1.64	114	853	ref.	—
1.64–2.01	148	813	1.37 (0.70–2.31)	1.96×10^–2^
2.01–2.56	211	846	1.87 (1.47–2.41)	6.25×10^–7^
>2.56	3148	836	28.20 (22.95–34.95)	<2.00×10^–16^

We used gender, age as covariates in this logistic regression models. ref.: reference. OR: odds ratio; 95%CI: 95% confidence interval.

**Fig 1:**
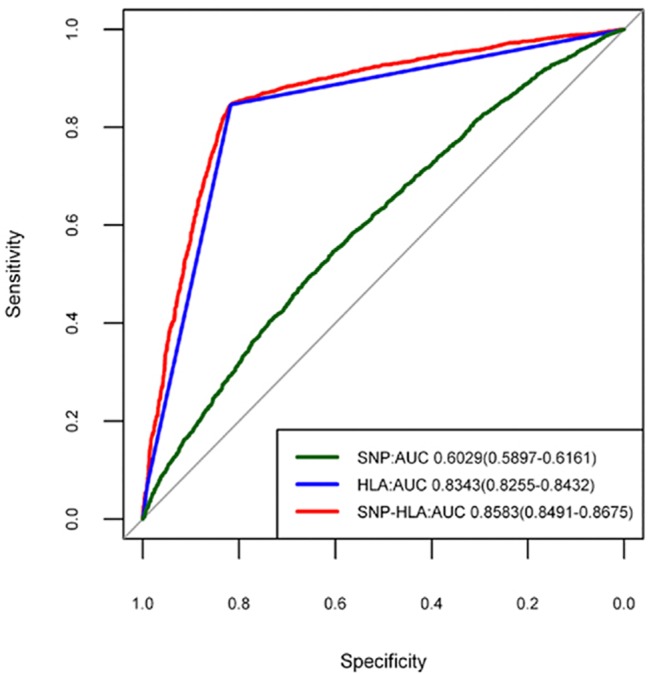
Receiver operating characteristic curves for predictive models in the initial stage.

**Fig 2:**
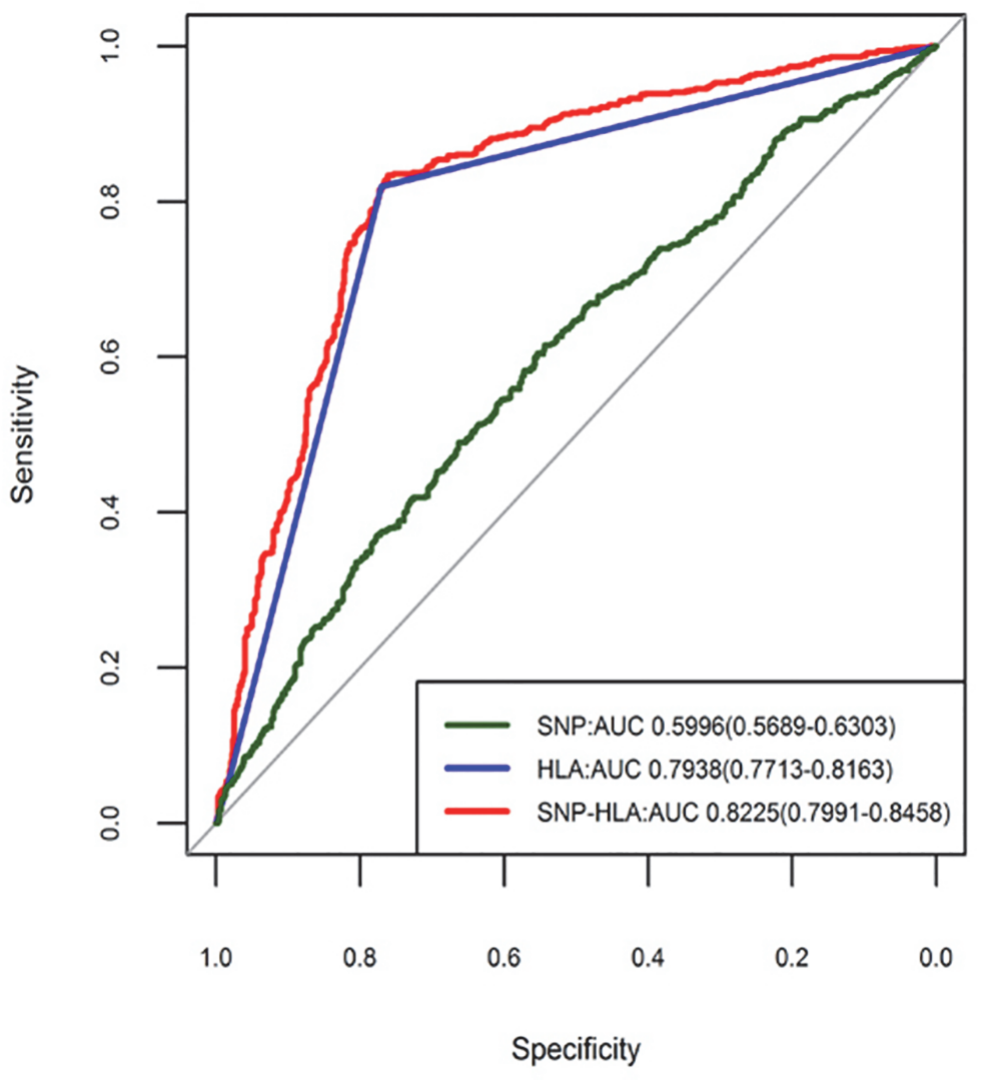
Receiver operating characteristic curves for predictive models in the replication stage.

We first built three different predictive models described in the Materials and Methods section using PRS (SNP model, HLA model and SNP-HLA model) in the samples from the initial stage. The SNP model had only a modest discriminatory effect for psoriasis (AUC = 0.6029, 95%CI: 0.5897–0.6161), suggesting that these SNPs confer only a small risk of psoriasis. However, the HLA model provided considerable discriminatory ability (AUC = 0.8343, 95%CI: 0.8255–0.8432), highlighting a dominant contribution of HLA locus to the risk of psoriasis. In the SNP-HLA model, we found the largest discriminatory ability (AUC = 0.8583, 95%CI: 0.8491–0.8675), which was significantly better (P < 2.20×10^–16^) than the HLA model (Fig 1). Furthermore, we found that the specificity and sensitivity of SNP-HLA model was quite high (Specificity = 81.70%, sensitivity = 84.70%), making this model possibly clinically useful. And in the validation samples, we got the consistent discriminatory results with those in the initial samples. It was also shown that the SNP-HLA model exhibited the highest discriminatory ability (AUC = 0.8225, 95%CI: 0.7991–0.8458), although the SNP model (AUC = 0.5996, 95%CI: 0.5689–0.6303) and HLA model (AUC = 0.7938, 95%CI: 0.7713–0.8163) both owned significant predictive abilities (Fig 2).

To evaluate the specificity of SNP-HLA PRS model for psoriasis, we assessed the ability of SNP-HLA PRS model in individuals with atopic dermatitis (ADerm) that has been previously described and consists of 1,012 affected individuals and 1,362 controls.[24] We found that the SNP-HLA PRS model had no ability to discriminate ADerm from healthy individuals (AUC = 0.51) whether we used PRS as a continuous or qualitative (i.e., “group”) predictor (S8 Fig).

### PRS and family history

In initial stage samples, we observed a significant difference in SNP-HLA PRS between cases with positive family history and cases with negative family history (PRS mean in cases with positive family history: 4.848 (s.d. = 1.356); PRS mean in cases with negative family history: 4.557 (s.d. = 1.416); P = 3.95×10^–9^). To explore the relationship between the PRS and self-reported family history, we tested the risk effect in three sets of ‘PRS’ categorization in our 3,621 cases from the initial stage, and found that PRS was elevated with a patient’s status of familial history (S5 Table). It was noted that 13 non-HLA SNPs, one HLA SNP and all together conferred similar risk effect, implying that these SNPs cumulatively increased the probability that the psoriasis would transmit from parents to offspring (P = 1.70×10^–2^–8.81×10^–9^).

### PRS and alcohol drinking

**Fig 3:**
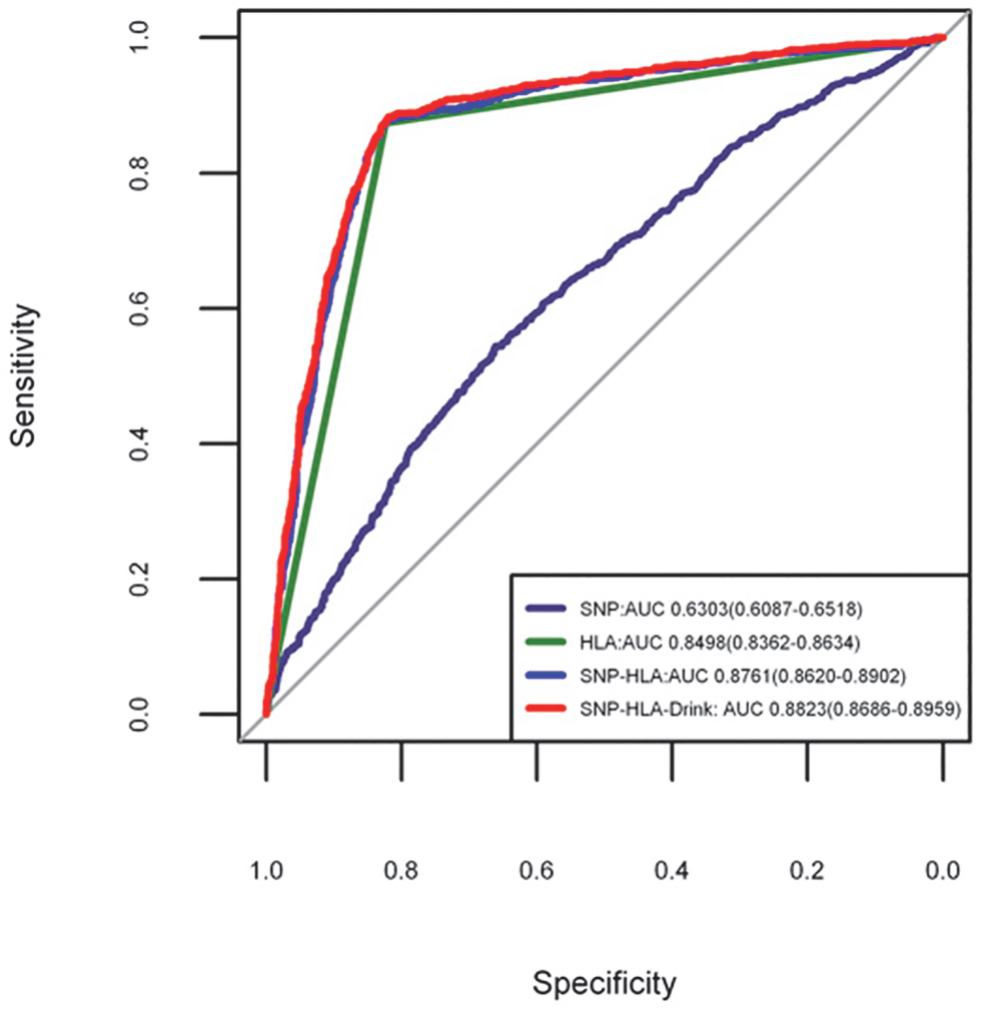
Receiver operating characteristic curves for predictive model incorporating alcohol drinking status.

There were 3,190 participants (882 cases and 2,308 controls) with alcohol drinking status available in the initial stage (S3 Table). We stratified our cohort based on SNP-HLA PRS values. And we observed none significant interaction between PRS group and alcohol drinking in these samples (P_interaction_ = 0.546) (S6 Table). We built an additional SNP-HLA-Drink model combined with drinking status, in order to test whether alcohol drinking status would improve the discriminatory accuracy. This analysis suggested that the AUC of SNP-HLA-Drink increased a little bit, but statistically significantly so (AUC = 0.885, 95%CI: 0.8716–0.8984; DeLong’s statistics for assessing model improvement: P = 2.82 ×10^–4^) (Fig 3).

### Cox proportional hazards test for age onset

**Fig 4:**
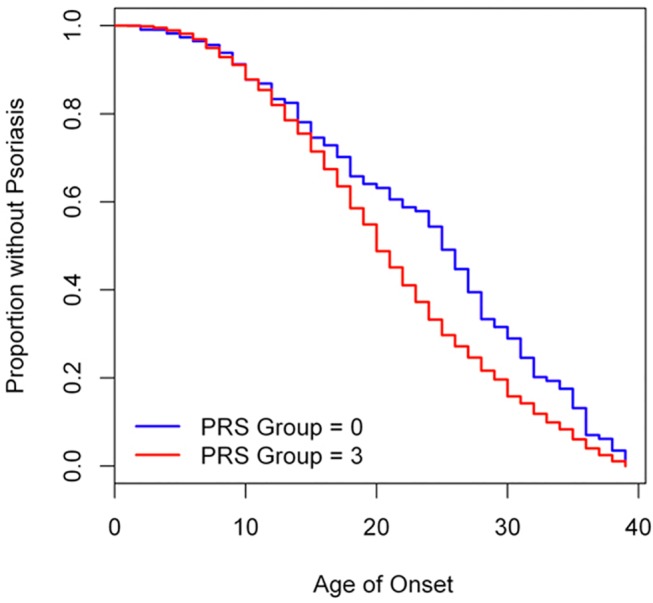
Kaplan-Meier curve: age of onset psoriasis stratified by SNP-HLA PRS groups.

**Fig 5:**
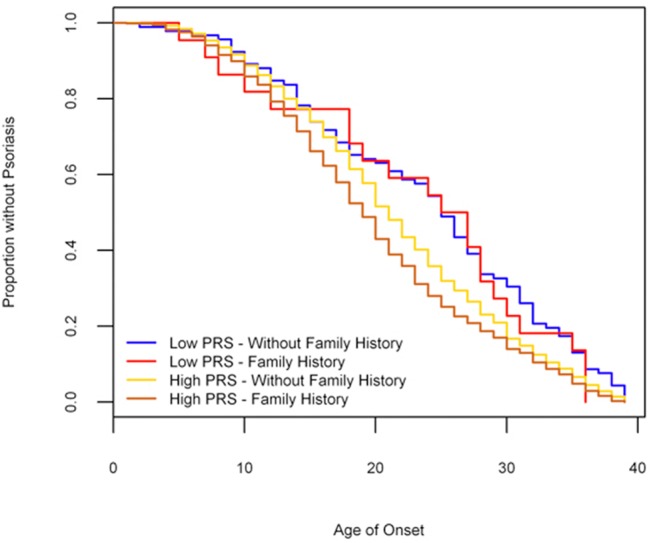
Kaplan-Meier curve: age of onset psoriasis stratified by SNP-HLA PRS group and family history.

We applied Cox proportional hazards tests to investigate whether PRS, family history and alcohol drinking were associated with age onset of psoriasis. In our 3,621 psoriasis cases with alcohol intake status available from the initial stage, it was noteworthy that significant pairwise differences in age onset existed among SNP—HLA PRS groups (F = 11.13, df = 3, P = 2.88×10^–7^). We also found that these SNPs cumulatively conferred a risk effect on the early onset of psoriasis (Hazard ratio (HR) = 1.08, P = 5.65×10^–12^) (S7 Table). In SNP-HLA PRS analysis, Kaplan-Meier curves of age of onset stratified by top quartile of PRS group (PRS group = 3) and bottom quartile of PRS group (PRS group = 0) showed an association of PRS group and age of onset (Log-rank = 12.6, df. = 1, P = 3.83×10^–4^) (Fig 4). The difference in median time to psoriasis (i.e., the time point at which half the cases have developed psoriasis) was five years between high risk group (PRS group = 3) and low risk group (PRS group = 0). We found that family history was significantly associated with early onset of psoriasis (HR = 1.20, P = 9.03×10^–7^) (S7 Table). Finally, we compared the Kaplan-Meier curves between SNP-HLA PRS high risk group (PRS group = 3) with family history and SNP-HLA PRS low risk group (PRS group = 0) without family history, and observed a significant difference of six years between the median age of onset between the two groups (Log-rank = 33.6, df = 3, P = 2.39×10^–7^, Fig 5).

## Discussion

We assessed 14 GWAS-implicated susceptibility variants for their cumulative effect on the risk and age onset of psoriasis, and present the predictive model with largest discriminatory accuracy to date in Han Chinese psoriasis. Our results highlight the potential of psoriasis GWAS findings in screening targeted populations.

In past decade, GWAS initiatives have been performed in psoriasis and ~ 40 susceptibility loci have been identified [4–12]. It gains much interest to incorporate these findings into genetic screening, with the ultimate goal to identify at-risk individuals in the general population for monitoring or intervention. However, collectively these genetic variants explain only a small component of the overall heritability of psoriasis [11, 13]. Furthermore, none of the identified variants has yet been used in medical diagnosis or indicating treatments. We built a predictive model using a weighted PRS encompassing 14 SNPs and evaluated its discriminatory ability. Our findings show the combined use of these 14 SNPs leads to reasonable discriminatory ability between psoriatics and non-psoriatics. Our predictive model exhibited an AUC of ~ 88%, the best predictive ability observed for a psoriasis predictive model to date. Moreover, our model has a sensitivity and specificity of around 80%. These results indicate that most of the predictive power of our models is provided by the HLA and the contributions of each individual non-HLA susceptibility variant and alcohol drinking are minimal but statistically significant none-the-less. We found that the HLA locus provides a major contribution to psoriasis risk, which is consistent with previous studies [25–27]. These findings are consistent with the estimation of psoriasis heritability conferred by different genetic components[3]. Our analyses suggest that the risk of psoriasis in high risk group increases ~ 28-fold compared with a low risk group. We confirmed the cumulative risk impact of these SNPs on psoriasis family history, which has been shown to be a significant predictor of psoriasis in a Caucasian population [19]. Moreover, we find that a combined SNP models can predict age onset of psoriasis. The result validates that the HLA region play the key role in this effect on age onset. Furthermore, our study found that the median age of onset for the high risk group was five years earlier than the low risk group.

It is noted that the predictive performance in samples from initial stage is higher than that in replication cohort. One possible reason accounted for this phenomenon is that all the samples in our initial stage have been used in our previous GWAS studies. And the SNPs we used in our predictive model were originally identified in our large discovery dataset. Therefore, the results of our modeling in the initial stage could be affected by over-fitting and a winner’s curse in the original data set. The predictive performance was evaluated and consistently validated in our independent replication cohort, suggesting the predictive models were potentially applicable. However, the predictive performance could be overestimated by sampling and the unique statistical models. Secondly, although ROC curves are useful to estimate the sensitivity and specificity of a model, they do not take into account the prevalence of disease in the population. The prevalence of psoriasis is estimated as 0.47% in Han Chinese population [28]. Therefore, although our models showed a high AUC of around 88%, which will likely prove informative, it may be of limited use in the general population.

In summary, we have shown the cumulative risk effect of the known genetic susceptibility variants on psoriasis susceptibility, and our results have produced the best predictive model in psoriasis to date, suggested the potential for genetic modeling in translational applications of GWAS findings.

## Supporting Information

S1 Fig The research design of our study. (TIF)

S2 Fig Distribution of SNP-HLA PRS between cases and controlsin the initial stage. (TIF)

S3 Fig Distribution of SNP-HLA PRS between cases and controlsin thereplication stage. (TIF)

S4 Fig Distribution of samples in diverse SNP-HLA PRS groups in the initial stage. (TIF)

S5 Fig Distribution of samples in diverse SNP-HLA PRS groups in the replication stage. (TIF)

S6 Fig Distribution of Odds Ratios of psoriasis risk by SNP-HLA PRS group in the initial stage. (TIF)

S7 Fig Distribution of Odds Ratios of psoriasis risk by SNP-HLA PRS group in the replication stage. (TIF)

S8 Fig The ROC curve of predictive HLA-SNP model in atopic dermatitis GWAS cohort. (TIF)

S1 Table The association results of 14 SNPs used in present and previous GWAS studies. (DOCX)

S2 Table The relationship between drinking and psoriasis used in our study. (DOCX)

S3 Table Characteristic of samples with alcohol abuse status available in the initial stage. (DOCX)

S4 Table The association results of 14 SNPs in our validation samples. (DOCX)

S5 Table The association between 3 sets of PRS and family history in psoriasis patients in the initial stage. (DOCX)

S6 Table The association of alcohol drinking stratified by PRS in the initial stage. (DOCX)

S7 Table Relationship between PRS, Family history, alcohol drinking and psoriasis age onset in the initial stage. (DOCX)
