# Supplementary material for: A Weighted Polygenic Risk Score Using 14 Known Susceptibility Variants to Estimate Risk and Age Onset of Psoriasis in Han Chinese
Source: PLoS One. 2015 May 1;10(5):e0125369. doi: 10.1371/journal.pone.0125369 (PMC4416725; doi:10.1371/journal.pone.0125369)
Supplement: S1 Table — (DOCX) [file pone.0125369.s009.docx]

**S1 Table : The association results of 14 SNPs used in present and previous GWAS studies**

| **SNP** | **A1** | **Multiple Analysis in Present study** | | **Published Results** | | | |
| --- | --- | --- | --- | --- | --- | --- | --- |
|  |  | **AF** | **OR(95%CI)** | **RAF** | **OR** | **Ncase** | **Ncontrol** |
| **rs151823^c^** | A | 0.5119782 | 1.20 (0.94-1.53) | 0.4943 | 1.12 | 7773 | 12095 |
| **rs3762999^c^** | G | 0.2448716 | 0.73 (0.41-1.31) | 0.233967 | 1.23 | 7773 | 12095 |
| **rs999556^c^** | A | 0.2554153 | 1.67 (0.94-2.98) | 0.237 | 1.25 | 7773 | 12095 |
| **rs7007032^c^** | C | 0.1948788 | 0.90 (0.63-1.29) | 0.177867 | 1.16 | 7773 | 12095 |
| **rs10088247^c^** | C | 0.1956678 | 1.35 (0.94-1.92) | 0.178733 | 1.17 | 7773 | 12095 |
| **rs3751385 ^c^** | T | 0.4985655 | 1.18 (1.02-1.37)* | 0.4734 | 1.18 | 4610 | 5373 |
| **rs514315^c^** | T | 0.7517573 | 1.16 (0.98-1.38) | 0.7343 | 1.15 | 7773 | 12095 |
| **rs9304742^c^** | T | 0.6705638 | 1.16 (0.99-1.35) | 0.6458 | 1.14 | 7773 | 12095 |
| **rs4085613^d^** | C | 0.6071582 | 1.35 (1.16-1.58)* | 0.5733 | 1.32 | 6860 | 8472 |
| **rs3213094^d^** | G | 0.5788266 | 1.38 (1.19-1.61)* | 0.5867 | 1.28 | 6860 | 8472 |
| **rs2431697^c^** | C | 0.1971023 | 1.27 (1.07-1.52)* | 0.17995 | 1.2 | 5749 | 6600 |
| **rs4649203^a^** | A | 0.6565772 | 1.23 (1.06-1.44)* | 0.6455 | 1.16 | 8399 | 10975 |
| **rs2303138^b^** | A | 0.4841486 | 1.00 (0.79-1.28) | 0.4573 | 1.16 | 8339 | 12725 |
| **rs1265181^d^** | G | 0.2845359 | 21.96 (17.79-27.29)* | 0.1 | 16.52 | 6206 | 7536 |

SNP: single nucleotide polymorphism. RAF: Risk allele frequency. RR: relative risk ratio. SE.: standard error. Ncase: number of cases. Ncontrol: number of controls. AF: allele frequency in full dataset.

In the multiple analysis, we used gender and age as covariates.* depicts the single-variant association p value < 0.05 in our full dataset.

^a^Li et al. Association analyses identifying two common susceptibility loci shared by psoriasis and systemic lupus erythematosus in the Chinese Han population. [J Med Genet.](http://www.ncbi.nlm.nih.gov/pubmed/24070858) 2013 Dec;50:812-8.

^b^Cheng et al. Identification of a Missense Variant in LNPEP that Confers Psoriasis Risk. [J Invest Dermatol.](http://www.ncbi.nlm.nih.gov/pubmed/?term=rs2303138+psoriasis)  2014 Feb;134:359-65.

^c^Sun et al. Association analyses identify six new psoriasis susceptibility loci in the Chinese population. [Nat Genet.](http://www.ncbi.nlm.nih.gov/pubmed/20953187) 2010 Nov;42:1005-9.

# ^d^Zhang et al. Psoriasis genome-wide association study identifies susceptibility variants within LCE gene cluster at 1q21. [Nat Genet.](http://www.ncbi.nlm.nih.gov/pubmed/19169255) 2009 Feb;41:205-10.
