# Supplementary material for: A Weighted Polygenic Risk Score Using 14 Known Susceptibility Variants to Estimate Risk and Age Onset of Psoriasis in Han Chinese
Source: PLoS One. 2015 May 1;10(5):e0125369. doi: 10.1371/journal.pone.0125369 (PMC4416725; doi:10.1371/journal.pone.0125369)
Supplement: S2 Table — (DOCX) [file pone.0125369.s010.docx]

**S2 Table: The relationship between drinking and psoriasis used in our study**

| **Factor** | **Exposed** | **RR** | **SE** |
| --- | --- | --- | --- |
| **alcohol^a^** | 0.43 | 1.531 | 0.137 |

Exposed: the proportion of ever-drinking in the general psoriasis cases.

RR: relative risk ratio. SE: standard error.

^a^Zhu et al. Alcohol consumption and psoriatic risk: a meta-analysis of case-control studies. [J Dermatol.](http://www.ncbi.nlm.nih.gov/pubmed/?term=Alcohol+consumption+and+psoriatic+risk%3A+a+meta-analysis+of+case-control+studies) 2012 Sep;39:770-3.
