# Supplementary material for: A Weighted Polygenic Risk Score Using 14 Known Susceptibility Variants to Estimate Risk and Age Onset of Psoriasis in Han Chinese
Source: PLoS One. 2015 May 1;10(5):e0125369. doi: 10.1371/journal.pone.0125369 (PMC4416725; doi:10.1371/journal.pone.0125369)
Supplement: S3 Table — (DOCX) [file pone.0125369.s011.docx]

**S3 Table: Characteristic of samples with alcohol abuse status available in the initial stage**

|  | **Case** | **Control** |
| --- | --- | --- |
| **No.** | 882 | 2308 |
| **Male (%)** | 519(58.84%) | 1339(58.02%) |
| **Age** |  |  |
| **mean(min-max)** | 30.57 (3 ~ 78) | 29.93(3 ~ 81) |
| **s.d.** | 11.86 | 10.84 |
| **Age onset** |  |  |
| **mean( min-max)** | 22.17(1~39) | -- |
| **s.d.** | 9.09 | -- |
| **Family History(%)** | 357(40.48%) | -- |
| **ever-drink** | 213(24.15%) | 284(12.31%) |

s.d.: standard deviation.

mean: the mean value of age/age onset.

min/max: the minimal/maximal vale of age/age onset.
