# Supplementary material for: A Weighted Polygenic Risk Score Using 14 Known Susceptibility Variants to Estimate Risk and Age Onset of Psoriasis in Han Chinese
Source: PLoS One. 2015 May 1;10(5):e0125369. doi: 10.1371/journal.pone.0125369 (PMC4416725; doi:10.1371/journal.pone.0125369)
Supplement: S4 Table — (DOCX) [file pone.0125369.s012.docx]

**S4 Table: The association results of 14 SNPs in our validation samples**

| **SNP** | **A1** | **Freq_control** | **OR** | **P** | **Power** |
| --- | --- | --- | --- | --- | --- |
| **rs4649203** | G | 0.34 | 1.01 | 0.86 | 5.36% |
| **rs4085613** | A | 0.43 | 0.77 | 6.53×10^-4^ | - |
| **rs151823** | C | 0.53 | 0.73 | 3.87×10^-5^ | - |
| **rs2303138** | A | 0.45 | 1.30 | 5.42×10^-4^ | - |
| **rs3762999** | G | 0.23 | 1.25 | 1.13×10^-2^ | - |
| **rs999556** | A | 0.24 | 1.24 | 1.21×10^-2^ | - |
| **rs3213094** | A | 0.45 | 0.80 | 3.25×10^-3^ | - |
| **rs2431697** | C | 0.19 | 1.07 | 0.51 | 10.21% |
| **rs1265181** | G | 0.13 | 5.24 | 5.27×10^-71^ | - |
| **rs7007032** | C | 0.20 | 1.16 | 0.11 | 37.23% |
| **rs10088247** | C | 0.20 | 1.09 | 0.33 | 16.16% |
| **rs3751385** | T | 0.48 | 1.09 | 0.23 | 22.16% |
| **rs514315** | C | 0.24 | 1.51 | 0.52 | 9.92% |
| **rs9304742** | C | 0.35 | 0.88 | 0.12 | 34.96% |

SNP: single nucleotide polymorphism. A1: the effective allele. Freq_control: the frequency of effective allele in controls. OR: odds ratio. Power: the statistic power at nominal significance (P<0.05) in 712 cases and 723 controls.
