# Supplementary material for: A Weighted Polygenic Risk Score Using 14 Known Susceptibility Variants to Estimate Risk and Age Onset of Psoriasis in Han Chinese
Source: PLoS One. 2015 May 1;10(5):e0125369. doi: 10.1371/journal.pone.0125369 (PMC4416725; doi:10.1371/journal.pone.0125369)
Supplement: S5 Table — (DOCX) [file pone.0125369.s013.docx]

**S5 Table: The association between 3 sets of PRS and family history in psoriasis patients in the initial stage**

| **PRS** | **OR(95%CI)** | **P values** |
| --- | --- | --- |
| **SNP** | 1.19(1.03-1.38) | 1.70×10^-2^ |
| **HLA** | 1.16(1.10-1.23) | 1.19×10^-7^ |
| **SNP-HLA** | 1.16(1.11-1.22) | 8.81×10^-9^ |

PRS: polygenic risk score by non-HLA SNPs, one HLA SNP or together. OR: odds ratio. 95%CI: 95% confidence interval.
