# Supplementary material for: A Weighted Polygenic Risk Score Using 14 Known Susceptibility Variants to Estimate Risk and Age Onset of Psoriasis in Han Chinese
Source: PLoS One. 2015 May 1;10(5):e0125369. doi: 10.1371/journal.pone.0125369 (PMC4416725; doi:10.1371/journal.pone.0125369)
Supplement: S6 Table — (DOCX) [file pone.0125369.s014.docx]

**S6 Table: The association of alcohol drinking stratified by PRS in the initial stage**

| **drink**  **polygenic**  **risk score** | **case** | | **control** | | **OR(95%)** | **P** |
| --- | --- | --- | --- | --- | --- | --- |
|  | **ever** | **never** | **ever** | **never** |  |  |
| **≤1.64** | 7 | 15 | 76 | 525 | 1.07(1.02-1.12) | 2.97×10^-3^ |
| **1.61-2.01** | 7 | 25 | 72 | 488 | 1.04(0.98-1.10) | 2.09×10^-1^ |
| **2.01-2.56** | 15 | 26 | 74 | 504 | 1.10(1.04-1.17) | 9.00×10^-4^ |
| **>2.56** | 184 | 603 | 62 | 507 | 1.25(1.16-1.34) | 3.14×10^-9^ |
| Test for Interaction^a^ | | | | | | 0.546 |

We included age and gender as covariates in each logistic regression model.

^a^LRT(3df)
