# Supplementary material for: A Weighted Polygenic Risk Score Using 14 Known Susceptibility Variants to Estimate Risk and Age Onset of Psoriasis in Han Chinese
Source: PLoS One. 2015 May 1;10(5):e0125369. doi: 10.1371/journal.pone.0125369 (PMC4416725; doi:10.1371/journal.pone.0125369)
Supplement: S7 Table — (DOCX) [file pone.0125369.s015.docx]

**S7 Table: Relationship between PRS, Family history, alcohol drinking and psoriasis age onset in the initial stage**

| **Component** | **HR(95%CI)** | **P** | **Sample size** |
| --- | --- | --- | --- |
| **SNP PRS** | 1.02(0.95-1.09) | 5.50×10^-1^ | 3621 |
| **HLA PRS** | 1.09(1.07-1.12) | 1.10×10^-12^ | 3621 |
| **SNP-HLA PRS** | 1.08(1.06-1.11) | 5.65×10^-12^ | 3621 |
| **Family History** | 1.20(1.11-1.28) | 9.03×10^-7^ | 3621 |
| **Alcohol drinking** | 0.85(0.72-1.01) | 5.87×10^-2^ | 882 |

PRS: polygenic risk score. HR: hazard ratio. 95%CI: 95% confidence interval.

In each Cox proportional hazard ratio model, we included age, gender as covariates. We used the continuous PRS to build the Cox model, while used none family history and never alcohol intake as reference.
